# Prostaglandin D_2_ Attenuates Bleomycin-Induced Lung Inflammation and Pulmonary Fibrosis

**DOI:** 10.1371/journal.pone.0167729

**Published:** 2016-12-19

**Authors:** Taiki Kida, Shinya Ayabe, Keisuke Omori, Tatsuro Nakamura, Toko Maehara, Kosuke Aritake, Yoshihiro Urade, Takahisa Murata

**Affiliations:** 1 Department of Animal Radiology, Graduate School of Agriculture and Life Sciences, The University of Tokyo, Tokyo, Japan; 2 International Institute for Integrative Sleep Medicine, University of Tsukuba, Ibaraki, Japan; Temple University School of Medicine, UNITED STATES

## Abstract

Pulmonary fibrosis is a progressive and fatal lung disease with limited therapeutic options. Although it is well known that lipid mediator prostaglandins are involved in the development of pulmonary fibrosis, the role of prostaglandin D_2_ (PGD_2_) remains unknown. Here, we investigated whether genetic disruption of hematopoietic PGD synthase (H-PGDS) affects the bleomycin-induced lung inflammation and pulmonary fibrosis in mouse. Compared with H-PGDS naïve (WT) mice, H-PGDS-deficient mice (*H-PGDS*^*-/-*^) represented increased collagen deposition in lungs 14 days after the bleomycin injection. The enhanced fibrotic response was accompanied by an increased mRNA expression of inflammatory mediators, including tumor necrosis factor-α, monocyte chemoattractant protein-1, and cyclooxygenase-2 on day 3. H-PGDS deficiency also increased vascular permeability on day 3 and infiltration of neutrophils and macrophages in lungs on day 3 and 7. Immunostaining showed that the neutrophils and macrophages expressed H-PGDS, and its mRNA expression was increased on day 3and 7 in WT lungs. These observations suggest that H-PGDS-derived PGD_2_ plays a protective role in bleomycin-induced lung inflammation and pulmonary fibrosis.

## Introduction

Pulmonary fibrosis is a progressive interstitial lung disease. It is characterized by increased deposition of extracellular matrix proteins as a result of proliferation and/or activation of fibroblasts. The architectural alteration in interstitial spaces ultimately leads to impairment of gas exchange and death. It is believed that transforming growth factor-β (TGF-β) plays a dominant role in the pathogenesis of pulmonary fibrosis by promoting proliferation and activation of fibroblasts and inducing epithelial-to-mesenchymal transition of epithelial cells. There has been little progress in treatment approach to directly modulate TGF-β signaling [1], and the fatality rate is still high. Therefore, understanding of more detailed mechanism of pulmonary fibrosis development is needed to provide therapeutic options other than organ transplantation to patients with this disease.

Inflammation is an important biological response against infection and tissue damage. Suppression or defect of inflammation fails to protect against infection and to heal wounds. On the other hand, chronic and/or excessive inflammation may lead to a variety of diseases from upper airway inflammation to cancer. Whereas idiopathic pulmonary fibrosis progresses without manifesting detectable inflammatory responses, many other forms of pulmonary fibrosis are associated with expression of inflammatory mediators such as tumor necrosis factor-alpha (TNF-α) [2], interleukin-1beta (IL-1β) [3], and interleukin-17 [4], and infiltration of inflammatory cells such as neutrophils, macrophages, and T cells [5]. Thus, inflammation is assumed to be crucial in progression of at least some types of pulmonary fibrosis [6].

Prostaglandins (PGs) are cyclooxygenase (COX)-dependent arachidonic acid metabolites that play crucial roles in inflammatory responses. PGs, particularly those mediated by COX-2 induction, are implicated in the pathogenesis of pulmonary fibrosis. PGE_2_ [7–11] and prostacyclin [12–14] have been shown to inhibit activation and proliferation of lung fibroblasts *in vitro* and *in vivo*. In contrast, PGF_2α_ is identified as an important mediator of pulmonary fibrosis by enhancing proliferation and collagen synthesis of lung fibroblasts through F-prostanoid receptor in a TGF-β-independent manner [15].

PGD_2_ is another COX metabolite which is synthesized by its specific enzymes. Hematopoietic PGD synthase (H-PGDS) is a cytosolic protein and responsible for PGD_2_ production in hematopoietic linage cells including mast cells [16] and Th2 lymphocytes [17]. PGD_2_ stimulates chemotaxis of eosinophils, basophils, and Th2 lymphocytes, resulting in enhanced inflammation [18]. PGD_2_ is also known as an inflammatory mediator of allergic asthma [19]. In contrast to these pro-inflammatory effects, PGD_2_ is reported to inhibit the activation of inflammatory cells such as antigen-specific T cells [20] and basophils [21]. PGD_2_ also inhibits tumor angiogenesis by suppressing vascular leakage and production of TNF-α [22]. The multifaceted actions of PGD_2_ in inflammatory processes are influenced by many factors such that expression level of its synthase, type of inflammatory stimuli, and the phase of the disease [23].

We have previously demonstrated that PGD_2_ inhibits inflammatory responses in endotoxin-induced acute lung injury including vascular hyper-permeability, immune cell infiltration, and cytokine production. Enhancement of anti-inflammatory PGD_2_ signal has proved to be beneficial in treating acute lung injury [24]. Inflammatory response including epithelial cell injury and subsequent infiltration of neutrophils and macrophages also plays an important part in triggering pulmonary fibrosis [25, 26]. These observations prompt us to investigate the roles of PGD_2_ in pulmonary fibrosis. Here, by using bleomycin-induced lung inflammation and pulmonary fibrosis model in mice, we revealed that PGD_2_ plays a protective role by suppressing inflammation.

## Materials and Methods

### Ethics Statement

All animal experiments were approved by the Institutional Animal Care and Use Committees of The University of Tokyo (approval no. p11-578) and performed according to the National Institute of Health guidelines. Mice were kept with irradiated food and bedding in the animal room with a light cycle of 12L/12D. Veterinary care and annual monitoring were under supervision of veterinary staffs dedicated to the facility. General anesthesia was induced with 4% isoflurane via a nose cone and continued with 2% isoflurane during procedures. The pedal withdrawal reflex was checked to ensure anesthetic depth, and all efforts were made to minimize suffering. For cardiac perfusion mice were euthanized with an overdose of anesthetic (sodium pentobarbital solution at least 200 mg/kg i.p.) followed by bilateral thoracotomy.

### Bleomycin-induced lung inflammation and pulmonary fibrosis model

*H-PGDS*^*-/-*^ mice (C57BL/6J background) were generated and bred as previously reported [27]. Mice were intratracheally instilled with bleomycin (1 mg/kg) in 50 μl saline. With this procedure, more than 80% of mice survived the 14-day duration of the current study. There was no significant difference in survival rate between WT mice and *H-PGDS*^*-/-*^ mice (S1 Fig).

### Hematoxylin-eosin staining and Masson trichrome staining

After lung was perfused with physiological salt solution containing heparin, lung tissues were excised, fixed in 4% paraformaldehyde for 3 days, and embedded in paraffin. Sections with 4 μm thickness were stained with hematoxylin-eosin or Masson trichrome.

### Immunohistochemistry

Lung tissues were fixed as described above, and embedded in OCT compound. Frozen sections with 4 μm thickness were permeabilized and blocked with 0.1% Triton X-100 and 5% normal goat serum for 30 min. Sections were then incubated with following primary antibodies overnight at 4°C: rabbit anti-H-PGDS antibody (1:1000), mouse anti-CD68 antibody (1:1000), and mouse anti-Gr-1 antibody (1:1000). After washing twice with PBS, sections were incubated with either secondary antibody, goat anti-rabbit IgG Alexa Fluor 488 or goat anti-mouse IgG Alexa Fluor 568 (1:500), for 3 h at room temperature. The sections were finally incubated with 4',6-diamidino-2-phenylindole (DAPI, 1 μg/ml) to stain the nuclei and photographed using a confocal fluorescence microscope (Eclipse Ti, Nikon, Japan) equipped with an argon laser. The number of Gr-1- or CD68-positive cells was counted in randomly selected fields.

### L-hydroxyproline content measurement

Lung homogenates in 1.5 ml HCl (2 mol/l) were incubated at 110°C for 16 h. Chloramine T solution (56 mmol/l chloramine T and 10% 1-propanol in citrate/acetate buffer) was added to the samples and incubated for 20 min. Ehrlich’s reagent (1 mol/l *p*-dimethylaminobenzaldehyde in 1-propanol and perchloric acid (2:1 ratio)) was added to the samples and incubated at 65°C for 20 min. The absorbance was measured at 550 nm by a spectrophotometer and L-hydroxyproline content was determined against a standard curve.

### Reverse transcription polymerase chain reaction (RT-PCR)

Total RNA was extracted from mouse lungs and reverse-transcribed into cDNA using random 9 mers and ReverTra Ace at 30°C for 10 min, 42°C for 60 min, and 99°C for 5 min. PCR amplification was performed using Ex Taq DNA polymerase and synthetic gene-specific primers shown in Table 1. After 30 cycles of amplification at 98°C for 10 sec, at 60°C for 30 sec, and at 72°C for 60 sec by a thermal cycler (Takara Bio, Japan), the PCR products were electrophoresed in a 2% agarose gel containing ethidium bromide (0.2 μg/ml). Detectable fluorescence bands were visualized using an ultraviolet transilluminator (Toyobo, Japan).

**Table 1:** Gene-specific primers.

Gene		Sequence
GAPDH	Sense	ACAGCAACTCCCACTCTTCC
	Antisense	GCCTCTCTTGCTCAGTGTCC
Col1a1	Sense	GAACCTGGTGATACTGGTGT
	Antisense	GAAGCCTCTTTCTCCTCTCTGAC
COX-2	Sense	CCCCCACAGTCAAAGACACT
	Antisense	CCCCAAAGATAGCATCTGGA
TNF-α	Sense	ACGGCATGGATCTCAAAGAC
	Antisense	CGGACTCCGCAAAGTCTAAG
MCP-1	Sense	CCCACTCACCTGCTGCTACT
	Antisense	AAGGCATCACAGTCCGAGTC
IL-1β	Sense	TGACGTTCCCATTAGACAGC
	Antisense	TGGGGAAGGCATTAGAAACA
IL-6	Sense	TCTCTGGGAAATCGTGGAAA
	Antisense	GATGGTCTTGGTCCTTAGCC
H-PGDS	Sense	CCTGGGCAGACTTCTACTGG
	Antisense	AAACTGCAACACCCCTTGAG

### Modified Miles assay

Evans blue dye (30 mg/kg) was intravenously injected in mice and circulated for 1 h. Lungs were then dissected and dried overnight at 55°C. After the lungs were weighed, Evans blue was extracted by incubation in formamide at 55°C overnight. The absorbance was measured at 610 nm by a spectrophotometer, and dye content was determined against a standard curve and normalized to lung dry weight.

### PGD_2_ measurement by LC-MS/MS

PGD_2_ levels in lung tissues were measured as described previously [28]. Specifically, excised lung was quickly frozen in liquid nitrogen and homogenized using Cryo-Press CP-50W (MICROTEC, Chiba, Japan). Tissues were then mixed with ethanol, centrifuged (800 g, 10 min), and the supernatant was diluted with 15% ethanol (in deionized water) containing 0.15% HCl (5 mol/l). PGD_2_-d4 was added as internal standard. The solutions were applied to Sep-Pak Vac 3 cc cartridges (Waters, Milford, MA). The cartridges were washed by n-hexane, and the samples were eluted with ethyl acetate and reconstituted in 10% acetonitrile (in deionized water). The samples were then applied to, and quantified by LCMS-8030 triple Quadrupole mass spectrometer (Shimadzu, Kyoto, Japan). Liquid chromatography was performed by using the Kinetex C18 column (Phenomenex, Torrance, CA).

### Chemicals

The chemicals used were as follows: bleomycin hydrochloride, heparin, L-hydroxyproline, paraformaldehyde, Triton X-100, formamide, *p*-dimethylaminobenzaldehyde, 1-propanol, and perchloric acid (Wako Pure Chemical, Japan); Evans blue and chloramine T (Sigma, MO); OCT compound (Sakura Finetek, Japan); DAPI (Dojindo Laboratories, Japan), random 9 mers and Ex Taq DNA polymerase (Takara Bio, Japan); ReverTra Ace (Toyobo, Japan); ethidium bromide, goat anti-rabbit IgG (H+L) Alexa Fluor 488 (#A-11008, Invitrogen, CA), and goat anti-mouse IgG (H+L) Alexa Fluor 568 (#A-11008, Invitrogen, CA); rabbit anti-H-PGDS polyclonal antibody (#O60760, Cayman Chemical, MI); mouse anti-CD68 monoclonal antibody (#MCA341R, AbD Serotec, UK); mouse anti-Gr-1 antibody (#108413, Biolegend, CA).

### Statistical analysis

The results are expressed as mean±S.E.M. Statistical evaluation of the data was performed by one-way analysis of variance followed by Bonferroni’s test for comparison among more than three groups, and by unpaired Student’s t test for comparison between two groups. A value of *P* <0.05 was taken as significant.

## Results

### H-PGDS deficiency accelerates bleomycin-induced lung inflammation and fibrosis

**Fig 1:**
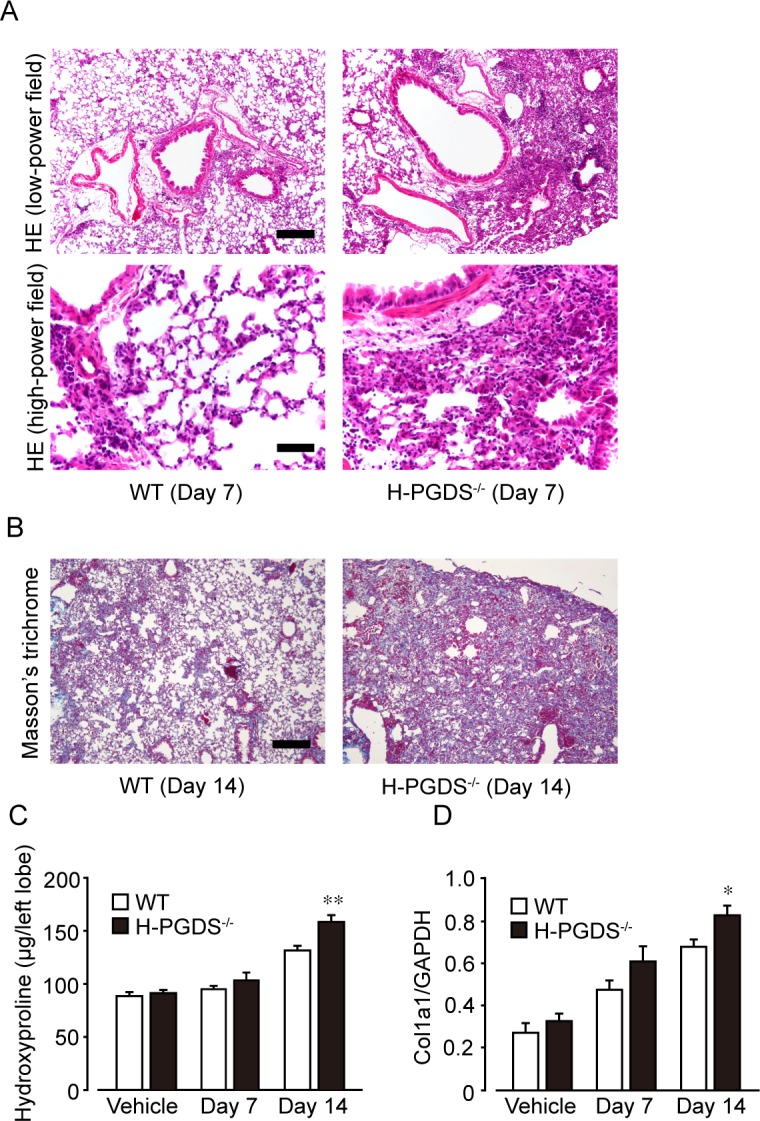
H-PGDS deficiency exacerbates bleomycin-induced pulmonary fibrosis in mice. (A) Representative pictures of hematoxylin-eosin (HE) staining on day 7. Scale bars, 400 μm (low-power) and 100 μm (high-power). (B) Typical sections of Masson’s trichrome staining of lung sections on day 14. Scale bar, 400 μm. (C and D) Hydroxyproline content (C) and Col1a1 mRNA expression (D) of the lungs after bleomycin treatment. *P < 0.05 and **P < 0.01 compared with WT mice (n = 4–10).

We first morphologically assessed the progression of inflammation and fibrosis in wild-type (WT) and H-PGDS deficient (*H-PGDS*^*-/-*^) mouse lungs. As shown in Fig 1A, intratracheal administration of bleomycin caused sporadic accumulation of inflammatory cells and alveolar wall thickening in lungs of both lines on day 7. *H-PGDS*^*-/-*^ mice had more severely distorted pulmonary architecture.

On day 14, both mice showed deposition of collagen fibers in lungs as indicated by Masson trichrome staining (Fig 1B). *H-PGDS*^*-/-*^ mice displayed more severe fibrotic response than WT mice did.

To quantitatively compare the fibrotic response in WT and *H-PGDS*^*-/-*^ mice, we measured hydroxyproline content in the lung tissues as an index of collagen accumulation. As shown in Fig 1C, administration of bleomycin increased the content of hydroxyproline in lungs and greater amount of hydroxyproline was detected in *H-PGDS*^*-/-*^ than that in WT mice on day 14. Consistently, mRNA expression of *Col1a1* in the lungs of *H-PGDS*^*-/-*^ mice was also greater than that of WT on day 14 (Fig 1D).

### H-PGDS deficiency increases early expression of inflammatory mediators in response to bleomycin

**Fig 2:**
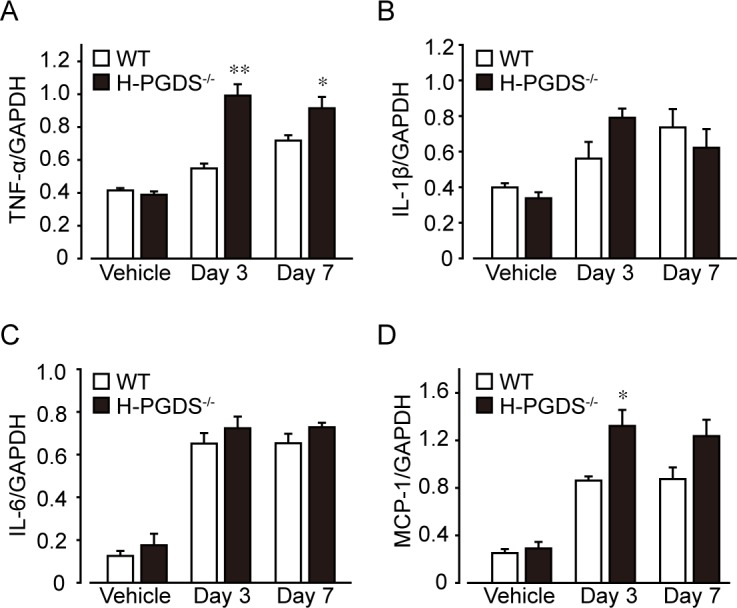
H-PGDS deficiency enhances early expression of inflammatory mediators. Total RNA was extracted from mouse lungs and mRNA expressions of TNF-α (A), Il-1β (B), Il-6 (C), and MCP-1 (D) were shown as the ratio of GAPDH. *P < 0.05 and **P < 0.01 compared with WT mice (n = 4).

We next assessed the mRNA expressions of inflammatory mediators in the bleomycin-treated mouse lungs. The administration of bleomycin increased expressions of TNF-α (Fig 2A), IL-1β (Fig 2B), interleukin-6 (IL-6, Fig 2C), and Monocyte chemoattractant protein-1 (MCP-1, Fig 2D) in both mouse lungs on day 3 and 7. H-PGDS deficiency accelerated the TNF-α expression on day 3 and 7 and MCP-1 expression on day 3. It also tended to increase IL-1β and IL-6 expressions on day 3 but the differences were not statistically significant. The result is consistent with our previous report that H-PGDS deficiency increased TNF-α production, resulting in enhanced inflammation and pro-tumorigenic environment [22].

### Neutrophils and monocytes/macrophages express H-PGDS in inflamed lung

**Fig 3:**
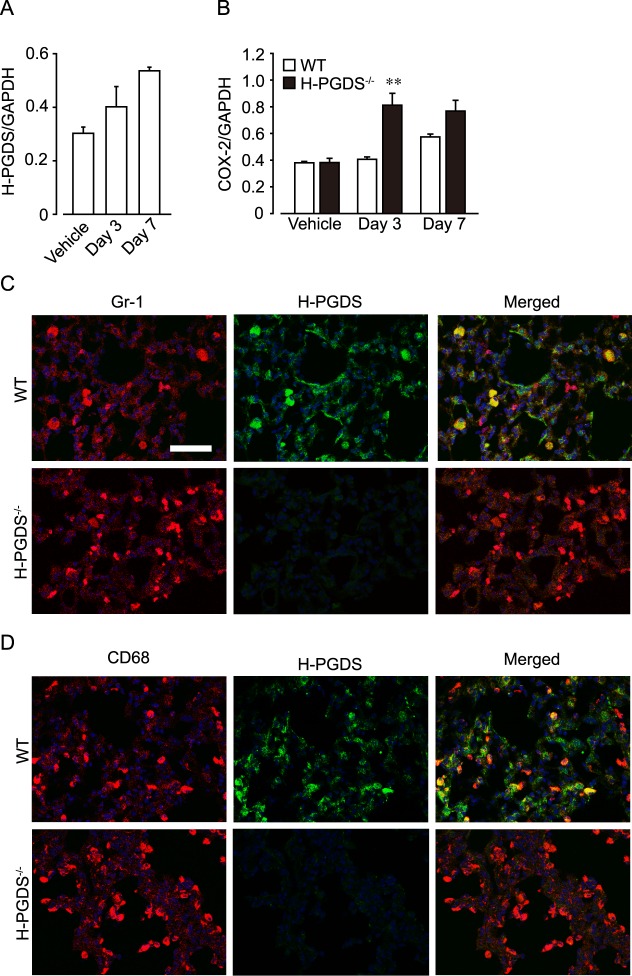
Infiltrating neutrophils and monocytes/macrophages express H-PGDS. mRNA expression of H-PGDS in WT mice (A) and COX-2 in WT and *H-PGDS*^*-/-*^ mice (B). **P < 0.01 compared with WT mice (n = 4). (C and D) Representative pictures of immunostained lung sections on day 7. H-PGDS expression was detected in neutrophils (C) and monocytes/macrophages (D). Nuclei were labeled with DAPI. Scale bar, 50 μm.

As shown in Fig 3A, the administration of bleomycin gradually increased mRNA expression of H-PGDS in WT mouse lungs on day 3 and 7. This response was accompanied with increased expression of COX-2 (Fig 3B). *H-PGDS*^*-/-*^ mouse lungs displayed greater COX-2 expression than WT on day 3, suggesting the increased inflammatory responses under H-PGDS deficiency.

8.45 ± 3.96 pg/mg of PGD_2_ was detected in WT mouse lungs, while 0.58 ± 0.38 pg/mg in *H-PGDS*^*-/-*^ mouse lungs on day 14.

Immunostaining showed that some Gr-1-positive neutrophils and CD68-positive monocytes/macrophages expressed H-PGDS in inflamed WT lungs on day 7. H-PGDS signal was absent in *H-PGDS*^*-/-*^ mice (Fig 3C and 3D).

### H-PGDS deficiency accelerates vascular permeability and infiltration of inflammatory cells

**Fig 4:**
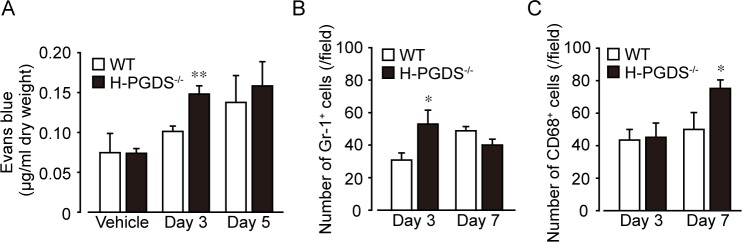
H-PGDS deficiency enhances bleomycin-induced vascular leakage and inflammatory cell infiltration in the mouse lung. (A) Evans blue dye was injected intravenously and circulated for 1 h. Dye contents were normalized to lung dry weight. **P < 0.01 compared with WT mice (n = 4–7). Number of Gr-1 positive (Gr-1^+^, B) and CD68 positive (CD68^+^, C) cells in the lung sections. *P < 0.05 compared with WT mice (n = 4–17).

We previously reported PGD_2_ inhibits inflammatory responses by suppressing vascular hyper-permeability an initial event of inflammation [22, 23]. Dye extravasation in the mouse lungs was measured as an index of vascular permeability (Fig 4A). Administration of bleomycin tended to increase dye extravasation in mouse lungs on day 3 and 5. *H-PGDS*^*-/-*^ mouse lung showed greater amount of extravasated dye in the lungs from on day 3 was significantly greater than that of WT mice (Fig 4A).

The number of infiltrated Gr-1-positive neutrophils was also greater in *H-PGDS*^*-/-*^ than in WT mice on day 3 (Fig 4B). On the other hand, the number of CD68-positive monocytes/macrophages in *H-PGDS*^*-/-*^ mice was greater than that of WT mice on day 7 (Fig 4C).

## Discussion

Previous studies have suggested the involvement of PGs in the pathogenesis of pulmonary fibrosis. As mentioned earlier, PGE_2_ [7–11] and prostacyclin [12–14] have an anti-fibrotic action, whereas PGF_2α_ is identified as a mediator of pulmonary fibrosis [15]. COX enzymes catalyze the arachidonic acid conversion to the intermediate prostaglandin PGH_2_, and specific prostanoid production is mediated by distal prostaglandin synthase enzymes. It is a powerful strategy to focus on temporal and spatial variation of each PG synthase expression to clarify the roles of each arachidonic acid metabolites.

In the present study, we demonstrated that *H-PGDS*^*-/-*^ mice showed increased fibrotic responses in the lung than WT mice 14 days after bleomycin administration (Fig 1). We next revealed that *H-PGDS*^*-/-*^ mice consistently displayed enhanced inflammation on day 3, which is characterized by increased expression of inflammatory mediators (Figs 2 and 3B) and neutrophil infiltrations (Fig 4B), vascular permeability (Fig 4A). Although disease stage-dependent action of PGD_2_ should be investigated in more depth, PGD_2_ may alleviate bleomycin-induced fibrosis partially by suppressing early inflammatory responses. Similarly, Riteau and Gasse *et al*. demonstrated that inhibition of extracellular ATP/P2X_7_ receptor [29] or uric acid/NALP3 inflammasome [30] signals suppresses lung inflammation as early as 24 h after bleomycin administration, which is followed by reduced fibrosis.

Immunostaining revealed that Gr-1 positive neutrophils and CD68-positive monocytes/macrophages strongly expressed H-PGDS in response to bleomycin challenge (Fig 3C and 3D). PGD_2_ produced in these types of cells may suppress infiltration of immune cells in an autocrine or a paracrine manner as suggested previously [24]. Epithelial cells and vascular endothelial cells are also shown to express H-PGDS in response to endotoxin. PGD_2_ from these resident lung cells is responsible for anti-inflammatory responses including suppression of vascular permeability in acute lung injury [24]. The relevance of the functional sources of PGD_2_ for anti-fibrotic responses warrants further study.

Besides anti-inflammatory actions, PGD_2_ might have direct effects on fibroblasts in the current model. Our group previously showed that PGD_2_ attenuates collagen secretion induced by TGF-β in human lung fibroblasts by activating D-prostanoid (DP) receptor [31]. Another group demonstrated that PGD_2_ inhibits proliferation of mouse lung fibroblasts via DP receptor [32]. We also investigated whether PGD_2_ is involved in apoptosis of fibroblasts. However, neither PGD_2_ (0.1–10 μM) nor a DP receptor agonist BW-245C (0.01–1 μM) altered the number of apoptotic cells under the 1 ng/mL TGF-β-stimulated condition assessed by TUNEL assay (S2 Fig).

Given its anti-inflammatory and anti-fibrotic actions discussed so far, enhancing the PGD_2_ signal can be a potential therapeutic option. A previous study demonstrated that transplantation of fibroblasts that constitutively express H-PGDS reduces bleomycin-induced lung injury, edema formation, and fibrosis in mice [33]. Further studies with the use of both genetic and pharmacological tools are required to evaluate the therapeutic potential of PGD_2_ signal enhancement in pulmonary fibrosis.

In summary, the present study suggests that PGD_2_ plays a protective role in bleomycin-induced pulmonary fibrosis, and the anti-fibrotic action of PGD_2_ can be partially explained by suppression of early inflammation.

## Supporting Information

S1 Fig Kaplan-Meier survival curves for bleomycin-exposed WT or *H-PGDS*^*-/-*^ mice. Kaplan-Meier survival curves for bleomycin-exposed WT (n = 27) or *H-PGDS*^*-/-*^ (n = 19) mice. There was no significant difference in survival rate (*P* = 0.484, log rank test) between WT (n = 27) and *H-PGDS*^*-/-*^ mice on day 14.
(DOCX)

S2 Fig The effect of PGD_2_ or BW-245C on IMR-90 apoptosis. The effect of PGD_2_ or BW-245C on IMR-90 apoptosis (n = 4). Treatment with TGF-β (1 ng/ml), PGD_2_ (0.1–10 μM), or BW-245C (0.01–1 μM) did not alter the number of apoptotic cells.
(DOCX)
