# Supplementary material for: Prostaglandin D2 Attenuates Bleomycin-Induced Lung Inflammation and Pulmonary Fibrosis
Source: PLoS One. 2016 Dec 19;11(12):e0167729. doi: 10.1371/journal.pone.0167729 (PMC5167321; doi:10.1371/journal.pone.0167729)
Supplement: S1 Fig — Kaplan-Meier survival curves for bleomycin-exposed WT (n = 27) or H-PGDS-/- (n = 19) mice. There was no significant difference in survival rate (P = 0.484, log rank test) between WT (n = 27) and H-PGDS-/- mice on day 14. (DOCX) [file pone.0167729.s001.docx]

**S1 Fig**

Kaplan-Meier survival curves for bleomycin-exposed WT (n = 27) or *H-PGDS^-/-^* (n = 19) mice. There was no significant difference in survival rate (*P* = 0.484, log rank test) between WT (n = 27) and *H-PGDS^-/-^* mice on day 14.
