# Supplementary material for: Prostaglandin D2 Attenuates Bleomycin-Induced Lung Inflammation and Pulmonary Fibrosis
Source: PLoS One. 2016 Dec 19;11(12):e0167729. doi: 10.1371/journal.pone.0167729 (PMC5167321; doi:10.1371/journal.pone.0167729)
Supplement: S2 Fig — The effect of PGD2 or BW-245C on IMR-90 apoptosis (n = 4). Treatment with TGF-β (1 ng/ml), PGD2 (0.1–10 μM), or BW-245C (0.01–1 μM) did not alter the number of apoptotic cells. (DOCX) [file pone.0167729.s002.docx]

**Supplementary Method**

**TUNEL assay**

Human fibroblast cell line IMR-90 (JCRB0516, JCRB cell bank, Osaka, Japan) was cultured in Dulbecco’s modified Eagle’s medium (DMEM) with 10% fetal bovine serum (FBS). After starving the cells in DMEM with 2% FBS for 48 h, TGF-β (1 ng/ml) and PGD_2_ (0.1-10 μM) or BW-245C (0.01-1 μM) was treated for 48 h, and TUNEL assay was performed using in situ TUNEL assay kit (Roche Diagnostics, Switzerland). DNase I (300 units/ml) was used as a positive control.

**S2 Fig**


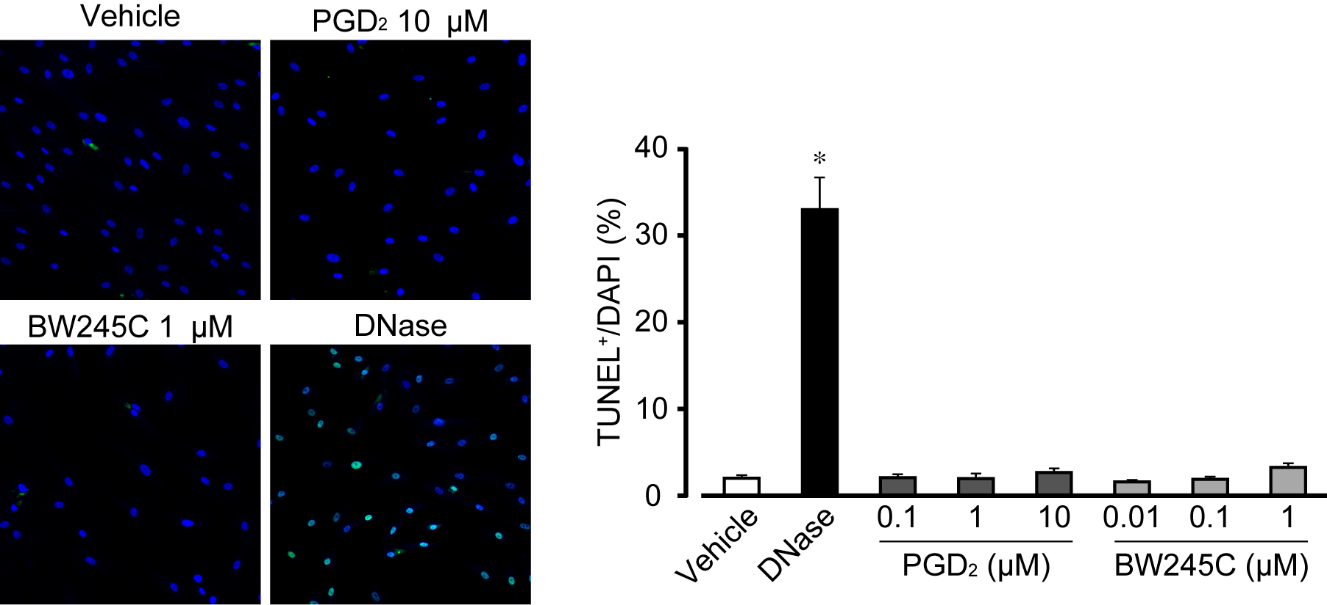


The effect of PGD_2_ or BW-245C on IMR-90 apoptosis (n = 4). Treatment with TGF-β (1 ng/ml), PGD_2_ (0.1-10 μM), or BW-245C (0.01-1 μM) did not alter the number of apoptotic cells.
